# Efficacy and Safety of Electroacupuncture for Pain Control in Herpes Zoster: A Systematic Review and Meta-Analysis

**DOI:** 10.1155/2022/4478444

**Published:** 2022-07-04

**Authors:** Kelin He, Fengjia Ni, Yi Huang, Mengyi Zheng, Han Yu, Dexiong Han, Ruijie Ma

**Affiliations:** ^1^Department of Acupuncture and Moxibustion, The Third Affiliated Hospital of Zhejiang Chinese Medical University, Hangzhou, Zhejiang, China; ^2^The Third School of Clinical Medicine (School of Rehabilitation Medicine), Zhejiang Chinese Medical University, Hangzhou, Zhejiang, China

## Abstract

**Introduction:**

Herpes zoster is caused by the reactivation of the latent varicella-zoster virus, which leads to acute pain that may disturb routine activities and affect patients' quality of life. Electroacupuncture (EA) has been commonly used for treating herpetic pain in clinical treatment. However, no relevant studies have been performed to evaluate the efficacy and safety of EA for acute control in herpetic neuralgia patients. The purpose of the current study was to conduct a systematic review and meta-analysis to address the deficiencies of the current research.

**Methods:**

Three English (PubMed, Cochrane Library, and Web of Science) and four Chinese (China National Knowledge Infrastructure (CNKI), Chinese Biomedical Literature database (CBM), Wan-fang database, and the Chinese Scientific Journals Full-text Database (VIP)) were comprehensively searched from inception to 31 December 2021. Two independent reviewers evaluated the retrieved data based on the eligibility criteria in advance. In addition, the Cochrane Risk of Bias Tool was used to assess the methodological quality of the included studies. Outcome indexes in this study included the visual analog scale, the time to cessation of pustules, the time to scabs, the time to rash healing, adverse reactions, and the incidence of postherpetic neuralgia. Sensitivity and subgroup analyses were also performed to evaluate the intervention effect specifically. In addition, publication bias was analyzed.

**Results:**

Six randomized controlled trials (167 participants in the experimental groups and 174 participants in the control groups) were identified as reporting the application of EA for acute herpes zoster pain and were included in this study. The results from our meta-analysis revealed that EA was superior to control treatment according to visual analog scale, the time of rash healing, and the incidence of postherpetic neuralgia. However, in terms of the time to cessation of pustules, scabs, and adverse reactions, the results showed that EA compared with the control group showed no significant difference. In addition, subgroup analyses indicated that 2/100 Hz-EA has more significant effects on herpetic pain. Sensitivity analyses revealed that the results of EA for acute pain control and the rash healing time in herpetic neuralgia patients were stable. However, a publication bias was observed.

**Conclusion:**

Our meta-analysis results showed that EA could offer certain advantages in treating acute pain in herpetic neuralgia patients. However, small sample sizes, heterogeneity in study design, and variable methodological quality weaken these inferences. In addition, weak evidence was found for the safety of EA.

## 1. Introduction

Acute herpes zoster pain is a feared disease caused by reactivation of the latent varicella-zoster virus located in the spinal or cranial sensory ganglia and usually occurs decades after the primary infection. It is mainly characterized by burning, shooting (like an electric shock), or intolerable pruritus in constant association with the outbreak of vesicular skin rash. [1–3] Moreover, these symptoms can severely influence the physical and mental health of patients, as well as their quality of life. An early study shows that herpes zoster commonly occurs in older patients, and herpes zoster-associated mortality increases with age. [4] Currently, early treatment with antiviral drugs such as acyclovir and vidarabine shortens the duration of skin lesions related to herpes zoster. [5] In terms of acute pain control in herpetic neuralgia patients, there is still no good management for treating this condition. Although nonsteroidal anti-inflammatory drugs (NSAIDs), antidepressants, and sympathetic nerve blockers are used to manage herpetic neuralgia, these treatments do not permanently alleviate severe pain. [6] But these drugs, even though effective, have more troubling adverse effects. In addition, early aggressive therapy is an important step forward for preventing postherpetic neuralgia. [7, 8] Therefore, developing new therapeutic strategies for treating acute pain in herpetic neuralgia patients is urgently needed.

As a vital part of complementary and alternative medicine, acupuncture has been widely applied in clinical practice. Previous studies have shown that acupuncture can treat various acute and chronic pain. [9, 10] Different acupuncture methods include manual acupuncture, electroacupuncture (EA), warm needling, auricular therapy, fire needling, etc., Currently, EA is one of the most common methods for treating pain in traditional Chinese medicine hospitals and has an excellent therapeutic effect on acute and chronic pain. [11–13] Recent studies have shown that EA can relieve pain by activating numerous bioactive chemicals through peripheral and central mechanisms and forestall the adverse impacts of often-debilitating pharmaceuticals. [14] Over recent years, some studies have confirmed that EA effectively relieves postherpetic neuralgia. [15, 16] However, the current state of evidence of EA for treating acute pain in herpetic neuralgia patients has been so far unknown. Therefore, this study aimed to answer these questions by conducting a systematic review and meta-analysis.

## 2. Methods

### 2.1. Design

This present study adhered to the Preferred Reporting Items for Systematic Reviews and Meta-Analyses (PRISMA) guidelines [17], and the study protocol has been registered on PROSPERO (Registration number: CRD42021297341).

### 2.2. Search Strategy

We had systematically searched the following seven electronic databases from inception to 31 December 2021: PubMed, Cochrane Library, Web of Science, CNKI, CBM, Wan-fang database, and VIP, to identify all the randomized controlled trials (RCTs) on EA for the treatment of acute pain in herpetic neuralgia patients. In addition, postgraduate theses or dissertations were also eligible. The following terms were searched as subject words, keywords, free-text terms, and MeSH terms: herpes zoster, shingles, herpetic neuralgia, acupuncture, acupuncture therapy, electroacupuncture. Apart from the above, there were no language, region, or countries restrictions.

### 2.3. Eligibility Criteria

This study included all available RCTs of EA for the treatment of acute pain in herpetic neuralgia patients. Any other types of literature such as system reviews, letters, case reports, editorials, animal studies, commentary, and non-RCTs were to be excluded.

### 2.4. Participants

Literature was included in which adult participants (older than 18 years) were diagnosed with herpetic neuralgia. All patients were in the acute phase of the disease (less than two weeks) and had not yet been treated.

### 2.5. Interventions

The intervention in the experimental group included EA alone or in combination with routine treatment (RT), and the control group included RT and/or sham EA.

### 2.6. Outcomes

The primary outcome indicator of this study was the pain severity, and the secondary outcome indicators included the time to cessation of pustules, the time to scabs, the time to rash healing, adverse reactions, and the incidence of postherpetic neuralgia.

### 2.7. Literature Selection and Data Extraction

One reviewer performed literature searches according to specified searching strategies and downloaded the related citations. All literature were imported into Endnote X9 software, and the duplicate literature was removed using electronic/manual checking. Subsequently, two independent reviewers screened and identified the titles and abstracts of the remaining literature, and then, independently retrieved the literature that fulfilled the inclusion criteria. Discussion or involving the corresponding author resolved any inconsistent result between reviewers. After initial screenings, two reviewers extracted data independently from the identified studies. The following information was extracted from each study: general information (authors, publish year), demographic data (sample size, intervention, age, sex), EA protocol (acupoints, acupuncture modality, retention time, and treatment duration), and outcome measure.

### 2.8. Data Analysis

#### 2.8.1. Assessment of Risk of Bias in Included Studies

Two independent reviewers evaluated the risk of bias of each study by using the Cochrane risk of the bias assessment tool. [18] This assessment tool mainly includes seven domains: random sequence generation, allocation concealment, blinding of participants and personnel, blinding of outcome assessment, incomplete outcome data, selective reporting, and other sources of bias. Each domain of the individual study was classified as high, low, or unclear risk. Discussions with the corresponding author resolved any discordance between the two reviewers.

#### 2.8.2. Statistical Analysis

All data analyses of this study were conducted with *R* software (version 3.6.3; package meta). Continuous variables were calculated as mean differences (MD) and at 95% confidence interval (CI). If the unit of MD varied between studies, standardized MD (SMD) was calculated. The random or fixed effects model was based on the clinical and methodological heterogeneity among the studies pooled in a meta-analysis. [19] The *I*^2^ statistic was used to evaluate the statistical heterogeneity of the studies (with *I*^2^ statistic > 50% indicating statistically significant heterogeneity). [20] In addition, sensitivity analyses and subgroup analyses were carried out to dissect the heterogeneity.

## 3. Results

### 3.1. Literature Selection

**Figure 1:**
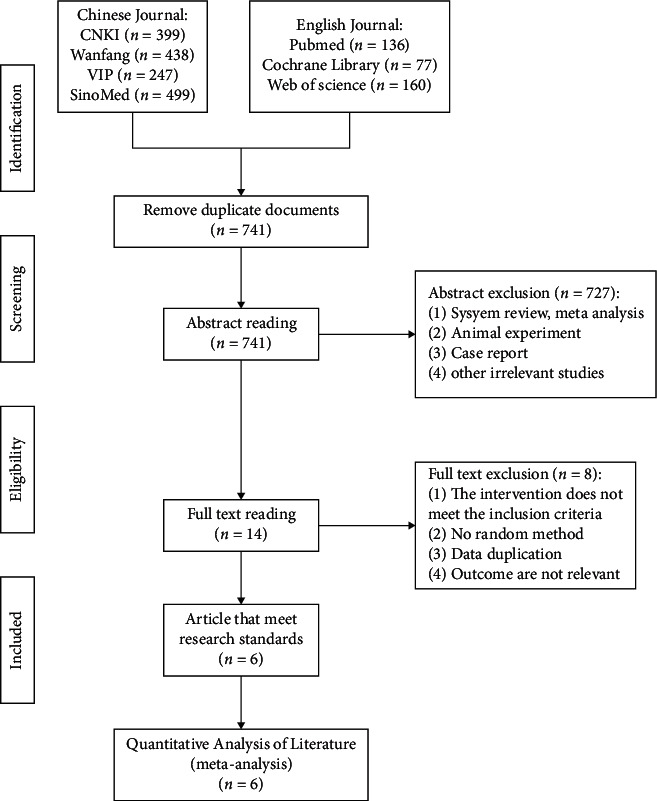
Flow diagram depicting the selection process of eligible studies. (CNKI, China National Knowledge Infrastructure; VIP, Chinese Scientific Journals Full-Text Database; SinoMed, the Chinese Biomedical Literature Database; *n* number of publications).

In total, 1956 published references were initially identified (399 references from CNKI, 438 references from Wan-fang, 247 references from VIP, 499 references from SinoMed, 136 references from PubMed, 77 references from Cochrane Library, and 160 references from Web of Science) and imported into Endnote X9. After eliminating duplicates, 741 articles were retained. We excluded reviews, case reports, animal experiments, and other irrelevant studies from these, and 14 studies remained. Moreover, mixed interventions, non-randomized methods, data missing, and outcome indicators that did not include outcomes were excluded. Finally, six studies were considered after full-text reading. The detailed flowchart of the literature screening process is shown in Figure 1.

### 3.2. Characteristics of Included Studies

A total of six articles were included, consisting of 341 participants with acute pain in herpetic neuralgia (*n* = 174 for the control group; *n* = 167 for the experimental group). The interventions in the control group included RT only, and the interventions in the experimental group were RT + EA or EA only. For outcome measure, six trials involved a visual analog scale, three involved times to cessation of pustules, scabs, and rash healing, and two reported adverse reactions and incidence of postherpetic neuralgia. The detailed characteristics of included studies are shown in Table 1.

### 3.3. Risk of Bias Assessment

**Figure 2:**
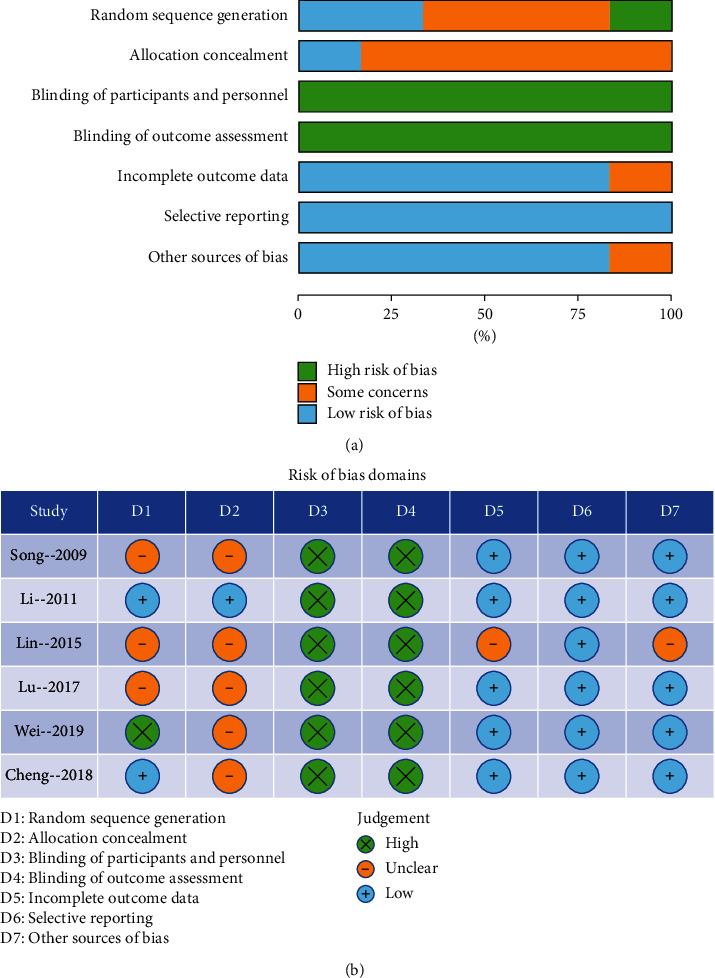
Bias risk assessment. (a) Risk of bias summary; (b) Risk of bias graph.

Figure 2 summarizes the risk of bias of the included studies. Regarding the random sequence generation, two trials [22, 26] reported the sequence generation method and were assessed as low risk of bias; three trials [21, 23, 24] only mentioned random but no specific method and was rated as unclear risk; one trial [25] did not mention randomization and was assessed as high risk. Concerning allocation, one study [22] provided the allocation concealment method in detail and was considered to be at a low risk of bias; five trials [21, 23–26] were rated as unclear risk of bias resulting from insufficient detail in the studies. For blinding of participants and personnel, six articles [21–26]were ranked as high risk of bias resulting from EA (a treatment) of procedural nature. Regarding the blinding of outcome assessments, six trials [21–26] were rated as high risk of bias because of no data regarding the assessment process. In terms of incomplete outcome data, five trials [21, 22, 24–26] recorded all results and were rated as low risk of bias; one trial [23] was unclear because they reported insufficient details to ensure that the baseline was balanced after dropping out. In terms of selective reporting, six studies [21–26] reported all data and were rated as low risk of bias. In addition, five trials [21, 22, 24–26] did not appear to any other potential sources and were assessed as low risk of bias; one article [23] was classified as an unclear risk due to insufficient details after patients dropped out of the trials.

### 3.4. Meta-Analysis Results

#### 3.4.1. The Pain Severity

**Figure 3:**
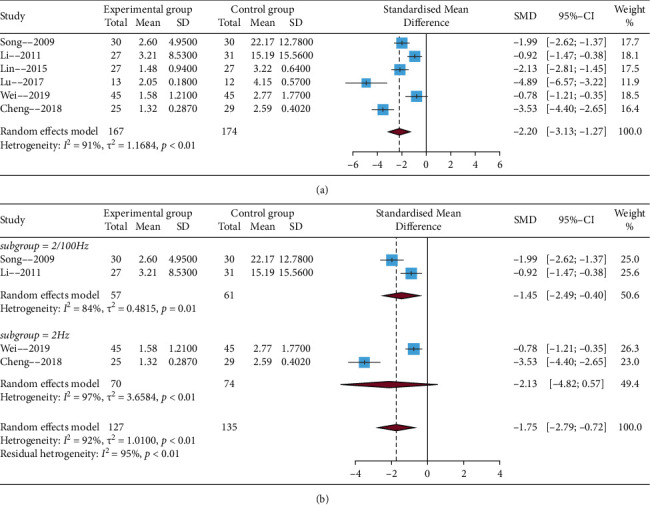
Funnel plot of the pain severity. (a) Standardized mean differences of VAS with experimental group compared with the control group. (b) Subgroup analyses.

All studies reported pain severity. After carefully reading the full text of corresponding studies, four trials used a visual analog scale (0–10 point), and two trials used another visual analog scale (0–100 point). Hence, SMD was calculated for the meta-analysis. The results of *I*^2^ statistic > 50%, the random-effect model was used to perform the meta-analysis. Results showed that EA compared with no EA showed a significant difference (SMD = −2.20, 95% CIs = −3.13; −1.27), which is presented in Figure 3(a). Subgroup analysis results showed that 2/100 Hz had a positive effect size (SMD = −1.45, 95% CIs = −2.49; −0.40) (Figure 3(b)). In addition, sensitivity analysis indicated that the results of this meta-analysis were reliable and robust after excluding studies one by one (details in Supplementary Material, FS1).

#### 3.4.2. The Cessation of Pustules Time

**Figure 4:**
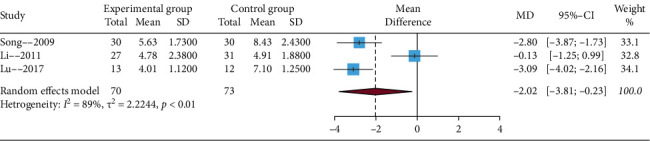
Funnel plot of the cessation of pustules time.

Among these studies, three studies involved the time to cessation of pustules. The definition of the cessation of pustules time is as follows: the time from the start of treatment until the blisters stop growing. Heterogeneity was significant (*I*^2^ statistic > 50%); therefore, the random effects model was used to perform the meta-analysis. The meta-analysis results showed that EA compared with no EA showed a significant difference (MD = −2.02, 95% CIs = −3.81; −0.23), which is presented in Figure 4. In addition, sensitivity analysis showed that the meta-analysis result was not stable. The sensitivity analysis was performed by sequentially deleting each original article. The results suggested that the main factors affecting the stability of outcomes were the studies conducted by Song [21] and Lu [24] (details in Supplementary Material, FS2).

#### 3.4.3. The Time to Scab

**Figure 5:**
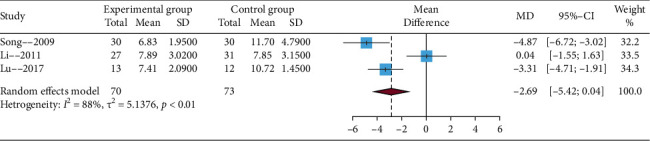
Funnel plot of the time to scab.

Among these studies, three studies reported the time to scabs. Heterogeneity was significant (*I*^2^ statistic > 50%), and random effects model was used to perform the meta-analysis. The results of this meta-analysis showed that EA compared with no EA showed no significant difference (MD = −2.69, 95% CIs = −5.42; 0.04), which is presented in Figure 5. In addition, sensitivity analysis revealed that the meta-analysis result was not stable. The sensitivity analysis was performed by sequentially deleting each original article. The results suggested that the main factors affecting the stability of outcomes were the studies conducted by Song [21] and Lu [24] (details in Supplementary Material, FS3).

#### 3.4.4. The Rash Healing Time

**Figure 6:**
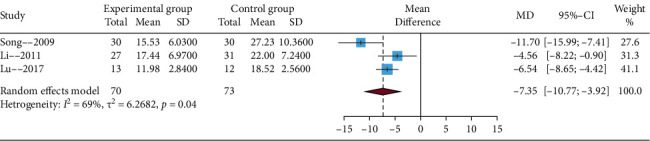
Funnel plot of the rash healing time.

Among these studies, only three trials provided the time to rash healing. Heterogeneity was significant (*I*^2^ statistic > 50%), therefore, the random effects model was applied. The results of this meta-analysis showed that EA compared with no EA showed a significant difference (MD = −7.35, 95% CIs = −10.77; −3.92), which is presented in Figure 6. In addition, sensitivity analysis showed that the results of this meta-analysis were credible (details in Supplementary Material, FS4).

#### 3.4.5. Safety Evaluation

**Figure 7:**
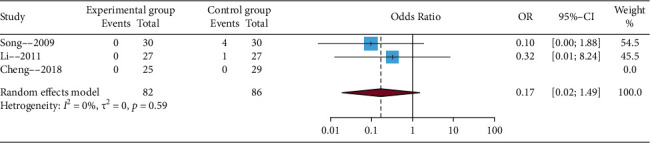
Funnel plot of the safety outcome.

Only three trials reported the clinical adverse events among these studies, including dizziness, gastrointestinal discomfort, and high fever. Considering potential clinical and methodological heterogeneity, even *I*^2^ statistic (statistical heterogeneity) < 50%, the random effects model was used to perform the meta-analysis. The results of this meta-analysis showed that EA compared with no EA showed no significant difference (OR = 0.17, 95% CIs = 0.02; 1.49), which is presented in Figure 7. In addition, sensitivity analysis showed that the results were not credible (details in Supplementary Material, FS5).

#### 3.4.6. The Incidence of Postherpetic Neuralgia

**Figure 8:**
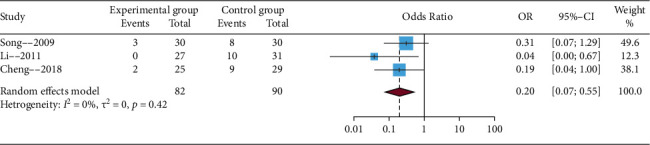
Funnel plot of the incidence of postherpetic neuralgia.

In our study, postherpetic neuralgia referred to pain in the lesion area after 1 month of herpes zoster. This result was in agreement with previous reports [27, 28]. Three trials reported the incidence of postherpetic neuralgia. Considering potential clinical and methodological heterogeneity, even *I*^2^ statistic (statistical heterogeneity) < 50%, the random effects model was used to perform the meta-analysis. The results of this meta-analysis showed that EA compared with no EA showed a significant difference (OR = 0.20, 95% CIs = 0.07; 0.55), which is presented in Figure 8. In addition, sensitivity analysis showed that the results were not credible (details in Supplementary Material, FS6).

### 3.5. Publication Bias

**Figure 9:**
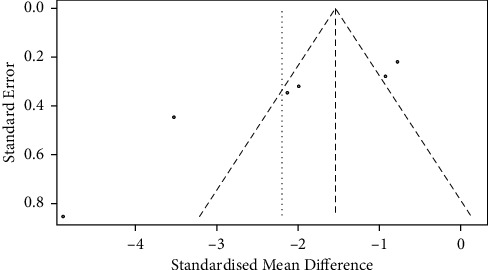
Meta-analysis results of publication bias.

Publication bias is a potential concern in meta-analyses when interpreting the results. In this study, the funnel plot and Begg's tests were used to assess the publication bias. [29] Publication bias was indicated by an asymmetry funnel around the pooled effect size. Here, it was worthwhile to notice that those studies lay not symmetrically around the pooled effect size, and the Begg's tests also revealed statistically significant publication bias (*p* < 0.05); the result is presented in Figure 9.

## 4. Discussion

Herpetic neuralgia is the most common and frequent clinical symptom after herpes zoster. Here, we launched a systematic review and meta-analysis to determine the efficacy and safety of EA for pain control in herpetic neuralgia patients. The present results indicated that EA was effective for pain control in herpetic neuralgia patients. Moreover, the time to rash healing and reducing the incidence of postherpetic neuralgia were remarkable. Nonetheless, only a minority of the studies have reported the adverse effect during the study; therefore, our study could not identify the safety of EA for pain control in herpetic neuralgia patients. Furthermore, only a small number of studies have reported the cessation of pustules and time to scabs in herpetic neuralgia patients; therefore, our meta-analysis could not determine the effectiveness of EA for the cessation of pustules and time to scabs. Overall, this is the first meta-analysis to conduct the study on efficacy and safety of EA for pain control in herpetic neuralgia patients. Hence, our study is very valuable; all details of this study are summarized below.

It is important to note that EA is effective for the treatment of acute pain in herpetic neuralgia patients. Previous studies suggest that EA is associated with reducing chronic pain, such as cervical myofascial pain syndrome and knee osteoarthritis. [12, 30] In addition, some studies indicate that EA is associated with reduced acute pain, including acute postoperative pain. [31] This study revealed that EA might also alleviate acute pain in herpetic neuralgia patients. Preclinical studies suggest that EA may lead to more substantial analgesic outcomes than manual acupuncture. [32] Moreover, it can decrease the risk of drug-drug interactions and the adverse effects of pharmaceutical drugs owing to their role in reducing the administration of analgesics. EA is defined as combining acupuncture and electric stimulation by inserting acupuncture into acupoints and passing a microcurrent close to human bioelectricity on the needle. [33] A previous study has shown that EA performed at different frequencies exhibits different analgesic effects. [12] In the present study, subgroup analysis found that 2/100 Hz-EA was better than 2 Hz-EA. The results obtained were consistent with the following studies: alternating low and high frequencies EA has a more potent analgesic effect than constant frequency EA. [34–36] In addition, for the acupoint of EA stimulation, the most commonly used points is Jiaji (EX-B2), followed by Zhigou (TE6), and Houxi (SI3). Jiaji (EX-B2) is located in the back region 0.5 inches lateral to the posterior median line. A previous study has shown that EA on Jiaji (EX-B2) can treat neuropathic pain. [37] Yet, as far as we know, no evidence for Zhigou (TE6) and Houxi (SI3) is observed.

It is also noteworthy that EA is effective for other symptoms and complications in herpetic neuralgia patients. First, the outcomes, indicator of skin lesions, including the rash healing time, pustules time, and scabs time, are commonly assessed in a clinical setting. In terms of the rash healing time, EA might also have a positive effect (MD = −7.35, 95% CIs = −10.77; −3.92). However, EA showed no positive effect in the cessation of pustules time and scabs time. Sensitivity analysis revealed that the meta-analysis result was not stable. Specifically, one study reported negative results [22], two studies reported positive results [21, 24]. The possible reason is that these outcomes mainly relied on the clinician's subjective judgment, which may easily lead to a detection bias. Due to this, more objective, precise, accurate, and reliable methods should be explored in daily clinical practice to identify skin lesions. In addition, this discrepancy may have been caused by the limited sample size. Second, in terms of the reduced incidence of postherpetic neuralgia, EA might have a positive effect (OR = 0.20, 95% CIs = 0.07; 0.55). It is generally known that postherpetic neuralgia is the most common intractable pain and seriously affects a patient's quality of life. In addition, it is very tricky to treat postherpetic neuralgia. Thus, effective prevention of postherpetic neuralgia is crucial for herpetic neuralgia patients. In our study, we found that EA might be a promising technique with a positive effect in the prevention of postherpetic neuralgia.

The safety of EA is also an important issue in herpetic neuralgia patients. Although EA can relieve acute pain in herpetic neuralgia patients and reduce the incidence of postherpetic neuralgia, we should also pay more attention to the safety aspects and adverse effects of EA. Regrettably, there is still a lack of evidence regarding the safety of EA in herpetic neuralgia patients. Only two of the six studies reported the adverse events, but the studies were underpowered to detect clinically significant differences in negative event rates. Several reasons for this are possible. First, the likely reason is that the sample size may be too small. Second, some researchers may believe that side effects were limited in severity and failed to report them. Although there are many studies with clear evidence of the safety of EA for treating pain [38], there is no convincing evidence concerning its safety in terms of EA for acute herpes zoster. Therefore, future studies should provide more details on the safety profile, regardless of favorable or unfavorable outcomes.

It is worth noting that the small sample sizes and poor methodological quality trials included in this review require attention. On the one hand, there were fewer studies, mostly with smaller sample sizes. In some trials, the sample size was as small as 12, and the largest trial had a sample size of 45. As a result, we detected potential publication bias cases using the observed funnel plot asymmetry. Therefore, to some extent, the small sample size limited the reliability of the estimated effects. On the other hand, there was remarkable heterogeneity between the studies regarding the intervention design. In particular, wide variation within the acupoints selected was observed. Since the efficacy of each acupoint may vary greatly, the pooled analysis results may not be generalizable to all included acupoint selection. In addition, EA as a procedural intervention was applied in the experimental group, and a similar procedural intervention was not conducted in the control group, the differences observed between the pooled experimental and control groups might be at least partially addressed by the differences in the placebo effect of these interventions. In addition, the sessions and courses of EA were not the same.

### 4.1. Limitation

There are some deficiencies in this study that need to be addressed. First, the sample size of the studies included in this view was relatively small. It is well-known that larger sample sizes may provide higher accuracy. Thus, we encourage authors to give the estimate sample size method using the statistical method. Second, the heterogeneity of study design of these studies was relatively high; for this reason, we encourage authors to register study protocols to improve the heterogeneity of experimental studies. Third, the methodological quality of these studies was relatively low. The lack of methodological quality among the included studies also limited the robustness of the results of this meta-analysis. Therefore, we encourage authors to precisely follow the Standards for Reporting Interventions in Clinical Trials of Acupuncture (STRICTA) guidelines.

## 5. Conclusion

Our results showed that EA could offer certain advantages in treating acute pain in herpetic neuralgia patients. However, small sample sizes, heterogeneity in study design, and variable methodological quality weaken these inferences. In addition, weak evidence was found for the safety of EA.

## Figures and Tables

**Table 1:** Characteristics of included studies.

Study	Publish year	Sample		Intervention		Age (years)		Sex (M/F)		Disease course (days)		Outcome measure
		*C*	*E*	*C*	*E*	*C*	*E*	*C*	*E*	*C*	*C*	
Song [21]	2009	30	30	RT: Valaciclovir, 300 mg, orally three times daily for ten days; vitamin B1, 10 mg, orally three times daily for ten days	EA: EA on the Jiaji (EX-B2) + Zhigou (TE6) + Houxi (SI 3); sparse and dense waves (2/100 Hz), treatment duration was 30 minutes, once a day for ten consecutive days.	43.47 ± 13.57	42.23 ± 14.98	15/15	16/14	4.77 ± 1.76	5.10 ± 1.45	VAS, the time to cessation of pustules, scabs, rash healing, the incidence of postherpetic neuralgia
Li et al. [22]	2011	31	27	RT: Valaciclovir, 300 mg, orally twice daily for ten days; vitamin B1, 10 mg, orally three times daily for ten days	EA: EA on the ashi points + Jiaji (EX-B2); Zhigou (TE 6) + Houxi (SI 3); sparse and dense waves (2/100 Hz), treatment duration was 30 minutes, once a day for ten consecutive days.	49.61 ± 16.34	48.39 ± 17.06	24/7	16/11	4.87 ± 2.25	5.25 ± 2.01	VAS, the time to cessation of pustules, scabs, rash healing, and the incidence of postherpetic neuralgia
Lin [23]	2015	27	27	RT: Indomethacin, 25 mg, three times daily for seven days; valaciclovir, 300 mg, orally twice daily for seven days; vitamin B1, 10 mg, orally three times daily for seven days	RT + EA: EA on the Hegu (LI4) + Waiguan (TE 5); Yanglingquan (GB34) + Zulinqi (GB41) + Taichong (LR3), continuous-wave (20 minutes for 80 Hz, 10 minutes for 20 Hz), treatment duration was 30 minutes, once a day for seven consecutive days.	18–45: 7 persons; 46–60: 6 persons; 60–80: 14 persons	18–45: 9 persons; 46–60: 8 persons; 60–80 : 10 persons	13/14	14/13	Less than one week	Less than one week	VAS
Lu [24]	2017	12	13	RT: Diclofenac, 75 mg, orally once daily for ten days; mecobalamin, 0.5 mg, orally three times daily for ten days; valaciclovir, 250 mg, orally three times for ten days; external 3% boric acid solution	RT + EA: EA on the Jiaji (EX-B2), continuous wave (60 Hz), treatment duration was 30 minutes, once a day for ten consecutive days.	47.14 ± 10.34	47.28 ± 10.41	5/7	6/7	Not mentioned	Not mentioned	VAS, the time to cessation of pustules, scabs, and rash healing
Wei [25]	2019	45	45	RT: Valaciclovir, 0.3 g, orally twice daily for 14 days; adenosylcobalamin, 0.5 mg, orally three times for 14 days; pregabalin, 150 mg, orally twice daily for 14 days	RT + EA: (EA on the Jiaji (EX-B2) + local points of rash, continuous-wave (2 Hz), treatment duration was 30 minutes, once a day for 14 consecutive days.	56.53 ± 9.15	57.89 ± 8.22	22/23	20/25	Less than one week	Less than one week	VAS
Cheng [26]	2018	29	25	RT: Valaciclovir, 0.3 g, orally twice daily for ten days; methylcobalamin, 0.5 mg, orally three times for ten days; If the pain were severe, oxycodone (10 mg) would be used.	RT + EA: EA on the Jiaji (EX-B2) + local ashi points, continuous-wave (2 Hz), treatment duration was 30 minutes, once a day for ten consecutive days	61.1 ± 2.13	51.40 ± 3.12	17/12	12/13	4.72 ± 0.38	4 ± 0.36	VAS, the incidence of postherpetic neuralgia

Abbreviations: C: control group; E: experimental group; VAS: visual analog scale.

## Data Availability

Data are available in a public, open access repository. All data relevant to the study are included within the article or uploaded as supplementary information.

## Supplementary Materials

Supplementary Materials Supplementary materials of sensitivity analysis results are available at Evidence-Based Complementary and Alternative Medicine online.
