# Supplementary material for: Efficacy and Safety of Electroacupuncture for Pain Control in Herpes Zoster: A Systematic Review and Meta-Analysis
Source: Evid Based Complement Alternat Med. 2022 Jul 4;2022:4478444. doi: 10.1155/2022/4478444 (PMC9273388; doi:10.1155/2022/4478444)
Supplement: Supplementary Materials — of sensitivity analysis results are available at Evidence-Based Complementary and Alternative Medicine online. [file 4478444.f1.pdf]

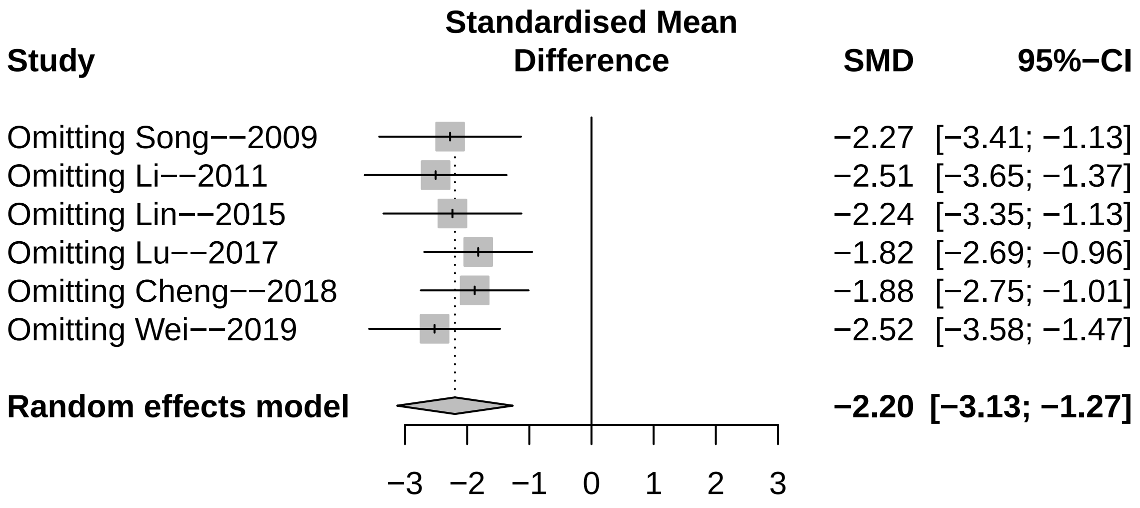

FS1,The pain severity

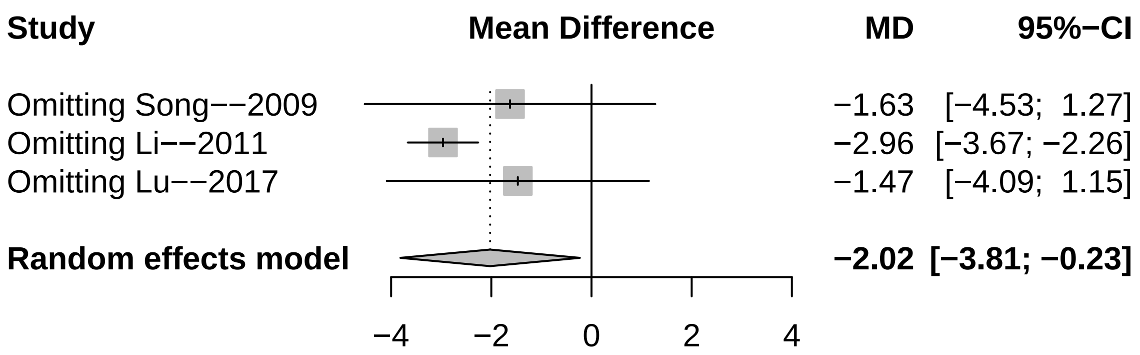

FS2, The cessation of pustules time

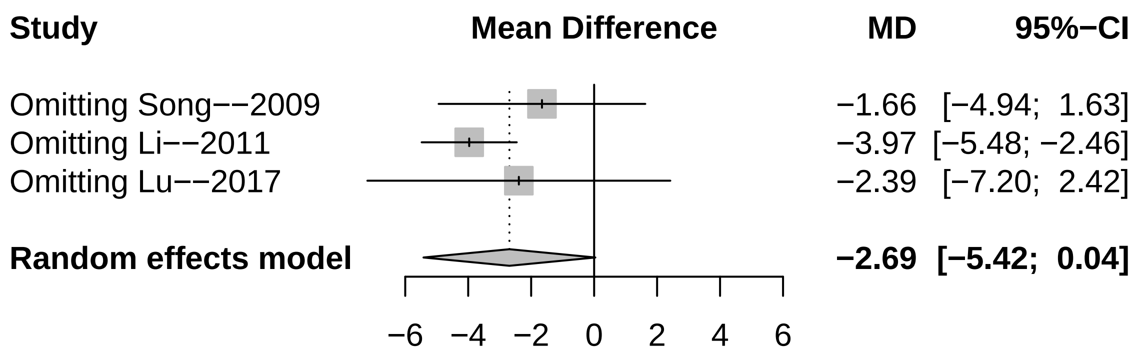

FS3, The time to scab

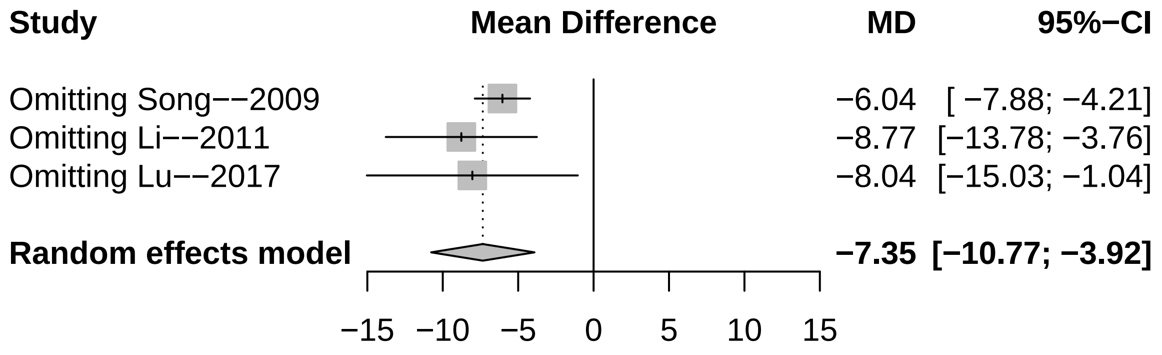

FS4, The rash healing time

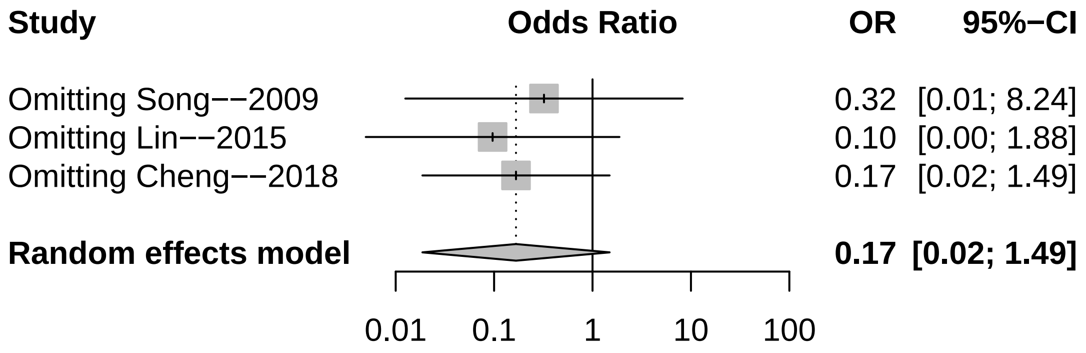

FS5, Safety evaluation

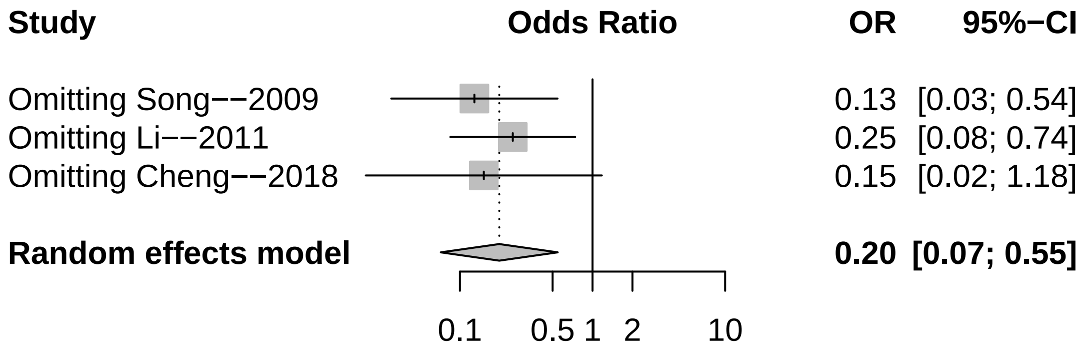

FS6, The incidence of postherpetic neuralgia
